# An *ADAMTSL2* Founder Mutation Causes Musladin-Lueke Syndrome, a Heritable Disorder of Beagle Dogs, Featuring Stiff Skin and Joint Contractures

**DOI:** 10.1371/journal.pone.0012817

**Published:** 2010-09-17

**Authors:** Hannah L. Bader, Alison L. Ruhe, Lauren W. Wang, Aaron K. Wong, Kari F. Walsh, Rebecca A. Packer, Jonathan Mitelman, Kathryn R. Robertson, Dennis P. O'Brien, Karl W. Broman, G. Diane Shelton, Suneel S. Apte, Mark W. Neff

**Affiliations:** 1 Department of Biomedical Engineering, Lerner Research Institute, Cleveland Clinic, Cleveland, Ohio, United States of America; 2 Veterinary Genetics Laboratory, University of California Davis, Davis, California, United States of America; 3 Department of Veterinary Clinical Sciences, Purdue University, West Lafayette, Indiana, United States of America; 4 Kingston Road Animal Hospital, Toronto, Ontario, Canada; 5 Department of Veterinary Medicine and Surgery, University of Missouri, Columbia, Missouri, United States of America; 6 Department of Biostatistics and Medical Informatics, University of Wisconsin, Madison, Wisconsin, United States of America; 7 Department of Pathology, School of Medicine, University of California San Diego, La Jolla, California, United States of America; Stanford University, United States of America

## Abstract

**Background:**

Musladin-Lueke Syndrome (MLS) is a hereditary disorder affecting Beagle dogs that manifests with extensive fibrosis of the skin and joints. In this respect, it resembles human stiff skin syndrome and the *Tight skin* mouse, each of which is caused by gene defects affecting fibrillin-1, a major component of tissue microfibrils. The objective of this work was to determine the genetic basis of MLS and the molecular consequence of the identified mutation.

**Methodology and Principal Findings:**

We mapped the locus for MLS by genome-wide association to a 3.05 Mb haplotype on canine chromosome 9 (*CFA9* (50.11–54.26; p_raw_ <10^−7^)), which was homozygous and identical-by-descent among all affected dogs, consistent with recessive inheritance of a founder mutation. Sequence analysis of a candidate gene at this locus, *ADAMTSL2,* which is responsible for the human TGFβ dysregulation syndrome, Geleophysic Dysplasia (GD), uncovered a mutation in exon 7 (c.660C>T; p.R221C) perfectly associated with MLS (*p*-value = 10^−12^). Murine ADAMTSL2 containing the p.R221C mutation formed anomalous disulfide-bonded dimers when transiently expressed in COS-1, HEK293F and CHO cells, and was present in the medium of these cells at lower levels than wild-type ADAMTSL2 expressed in parallel.

**Conclusions/Significance:**

The genetic basis of MLS is a founder mutation in ADAMTSL2, previously shown to interact with latent TGF-β binding protein, which binds fibrillin-1. The molecular effect of the founder mutation on ADAMTSL2 is formation of disulfide-bonded dimers. Although caused by a distinct mutation, and having a milder phenotype than human GD, MLS nevertheless offers a new animal model for study of GD, and for prospective insights on mechanisms and pathways of skin fibrosis and joint contractures.

## Introduction

The critical role of extracellular matrix (ECM) in sequestration and regulated release or activation of growth factors is highly relevant to common medical problems such as fibrosis. An excellent example showcasing this role is regulation of TGF-β by tissue microfibrils, which contain the glycoprotein fibrillin-1 as a major constituent. The three TGFβ isoforms exist as disulfide-bonded dimers [1]. Their propeptides are processed intracellularly by furin, but remain associated with the active dimer (the latency-associated peptide) [1] as the small latent complex (SLC), which in turn, binds to latent TGFβ–binding protein (LTBP)-1, -3, and -4, to form a large latent complex (LLC) [1], [2], [3]. The LLC is sequestered in the ECM through binding of LTBP1 to the ECM glycoproteins fibrillin-1 and fibronectin [4], [5], [6], [7]. TGFβ activity appears to be regulated primarily at the level of activation in ECM, which occurs either by a non-proteolytic mechanism involving integrins and thrombospondin-1, or *via* proteolysis of the LLC [1]. Because of the critical role of TGFβ in common medical disorders, even rare genetic disorders that reveal mechanisms of its regulation are potentially significant.

Fibrillin-1 mutations have clinically diverse consequences, since they can result in Marfan syndrome (MFS) [8], an acromelic dysplasia named Weill-Marchesani syndrome (WMS) [9], stiff skin syndrome (SSS) [10] and isolated ectopia lentis [11]. In MFS, fibrillin-1 haploinsufficiency is believed to lead to inadequate retention of the LLC, giving rise to inappropriate TGFβ activity in the lung, heart valves and aortic root [12], [13], [14], [15]. Recently, domain-specific mutations affecting fibrillin-1 were reported in SSS, the cardinal manifestation of which is skin fibrosis and restricted joint movement [10]. Clinical and mechanistic analysis of this rare disorder demonstrated similarities with scleroderma, a hardening of skin, which typically occurs in adults as an acquired disorder [10]. The *Tight skin (Tsk)* mouse is caused by an in-frame, partial duplication of *Fbn1* [16], [17], [18]. Thus, there is a strong link between fibrillin-1 and skin fibrosis.

Another rare disorder in which skin thickening and joint contractures are characteristic features, is geleophysic dysplasia (GD), an acromelic dysplasia in which short stature, characteristic facies, a pleasant temperament, shortening of the distal phalanges, and tracheal stenosis are also present [19]. GD results from recessively inherited *ADAMTSL2* mutations [20]. The medium of GD fibroblasts was found to contain high levels of TGFβ, and GD fibroblasts were shown to have evidence of enhanced TGFβ signaling [20]. ADAMTSL2 binds LTBP1 [20], whose domain structure resembles the fibrillins, although direct association between ADAMTSL2 and fibrillin-1 has not yet been reported. The related acromelic dysplasia, WMS, resembles GD, in having stiff, thick skin, and stiff joints. However, GD is distinctive in having severe cardiopulmonary involvement, and often leads to childhood mortality [19], [20]. In contrast to GD, patients with WMS suffer dislocation of the lens because of a defective zonule, severe glaucoma, and extreme vision impairment not seen in GD [21]. In addition to dominant *FBN1* mutations [9], WMS is caused by recessively inherited *ADAMTS10* mutations [22], [23], [24]. The recent discovery of *ADAMTS17* and *ADAMTSL4* mutations in a WMS-like syndrome [24] and in recessive isolated ectopia lentis [25] respectively, further strengthen genetic associations between fibrillin-1 microfibrils and the ADAMTS superfamily. This superfamily contains secreted ADAMTS metalloproteases as well as ADAMTS-like proteins, which are not proteases, but secreted glycoproteins whose domain structure resembles the C-terminal ancillary domains of ADAMTS proteases [26]. In addition to these genetic associations, ADAMTSL6 was recently shown to bind fibrillin-1 and to accelerate fibrillin biogenesis in vitro, and in transgenic mice overexpressing this protein [27]. Thus, several lines of genetic or experimental evidence suggest a functional link between members of the ADAMTS superfamily and fibrillin-1 microfibrils or fibrillin-1 binding proteins such as LTBP1.

Although many hereditary disorders in man have canine counterparts, no TGFβ dysregulation syndromes have yet been identified in the dog. One canine syndrome, originally called “Chinese beagle syndrome” in the lay literature, and subsequently renamed after two noted beagle breeders, Musladin and Lueke, is characterized by short stature, thick, taut skin, and severely restricted joint mobility, thus resembling SSS, GD and WMS in these aspects. Affected dogs also have broad skulls with wide-set slanted eyes, creased ears, a hopping, “tip-toe” gait, and pleasant temperaments. MLS was first reported in Beagles in the 1970s, with an incidence of 2–3% in British and Australian subpopulations. Musladin and Lueke suggested the disorder was a Mendelian defect segregating with autosomal recessive inheritance [28]. Here, the genetic and molecular basis of MLS is described. We identified a missense founder mutation in *ADAMTSL2* that causes MLS, and have found that the mutation results in the formation of anomalous disulfide-bonded dimers.

## Results

Previous descriptions of MLS phenotypes were verified by interviews with owners and clinical examination of affected dogs. As puppies, affected dogs failed to thrive, showed stunted growth, and reportedly suffered “phantom” bouts of pain. The condition appeared to stabilize by a year of age, though arthritis was a common sequel. Affected dogs consistently had short outer digits, reminiscent of the outer phalanx that is characteristic of children with Down syndrome. Owners also reported a high-pitched bark and a particularly ‘gregarious’ temperament. Interestingly, a ‘happy’ temperament is also a hallmark of children with GD. Seizures were noted in several affected dogs.

**Figure 1:**
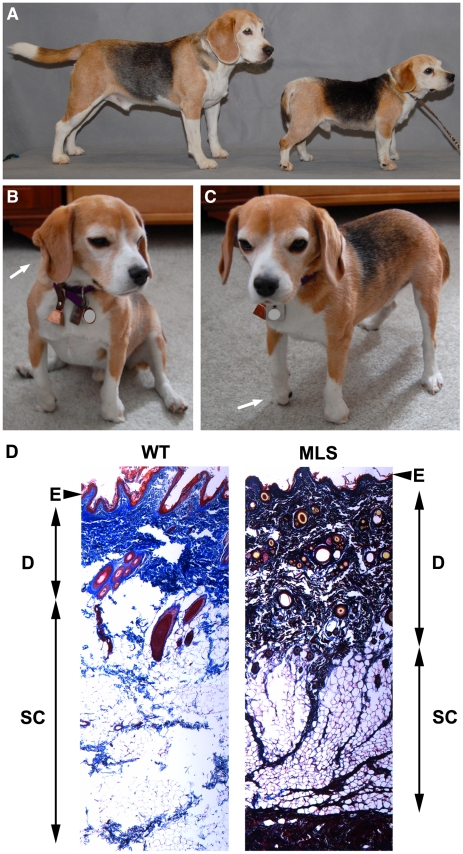
Phenotypic presentation of MLS in Beagle dogs. (**A**) Non-affected (left) and affected (right) Beagles matched by age, sex, and variety. Note short stature of affected dog. (**B**) Affected dog showing ear crease (arrow) and unusual stiff sitting posture. (**C**) Same dog as in B showing hyper-digitigrade standing (arrow), which is also noticeable in panel B. The typical facial appearance is seen in B and C. (**D**). Histology of skin from an MLS case compared to skin from an unaffected dog (WT). Stain: Masson trichrome (collagen = blue). Note the increased intensity and extent of blue staining corresponding to collagen and the presence of collagen strands extending from the dermis to underlying tissue in MLS skin. E, epidermis, D, dermis, SC, subcutaneous fat.

Clinical assessment of two affected dogs (**Text S1**) revealed thick, inelastic skin, and stiffness of the limbs, which could not be put through a full range of motion, especially at the distal joints (**Figure 1A**–**C**). The stiffness persisted even under a general anesthetic. Necropsy of an affected MLS puppy did not reveal any abnormality in organ systems other than thick skin (**Figure 1D**), which was adherent to underlying structures, and dense craniofacial bone with normal dentition. The linea alba of the ventral abdominal wall, a well-defined fibrous structure, was roughly five times the normal width. Because MLS has not been described previously in the veterinary literature, clinical details of these cases are summarized as supplemental information (**Text S1**).

### Genetic mapping of MLS

**Figure 2:**
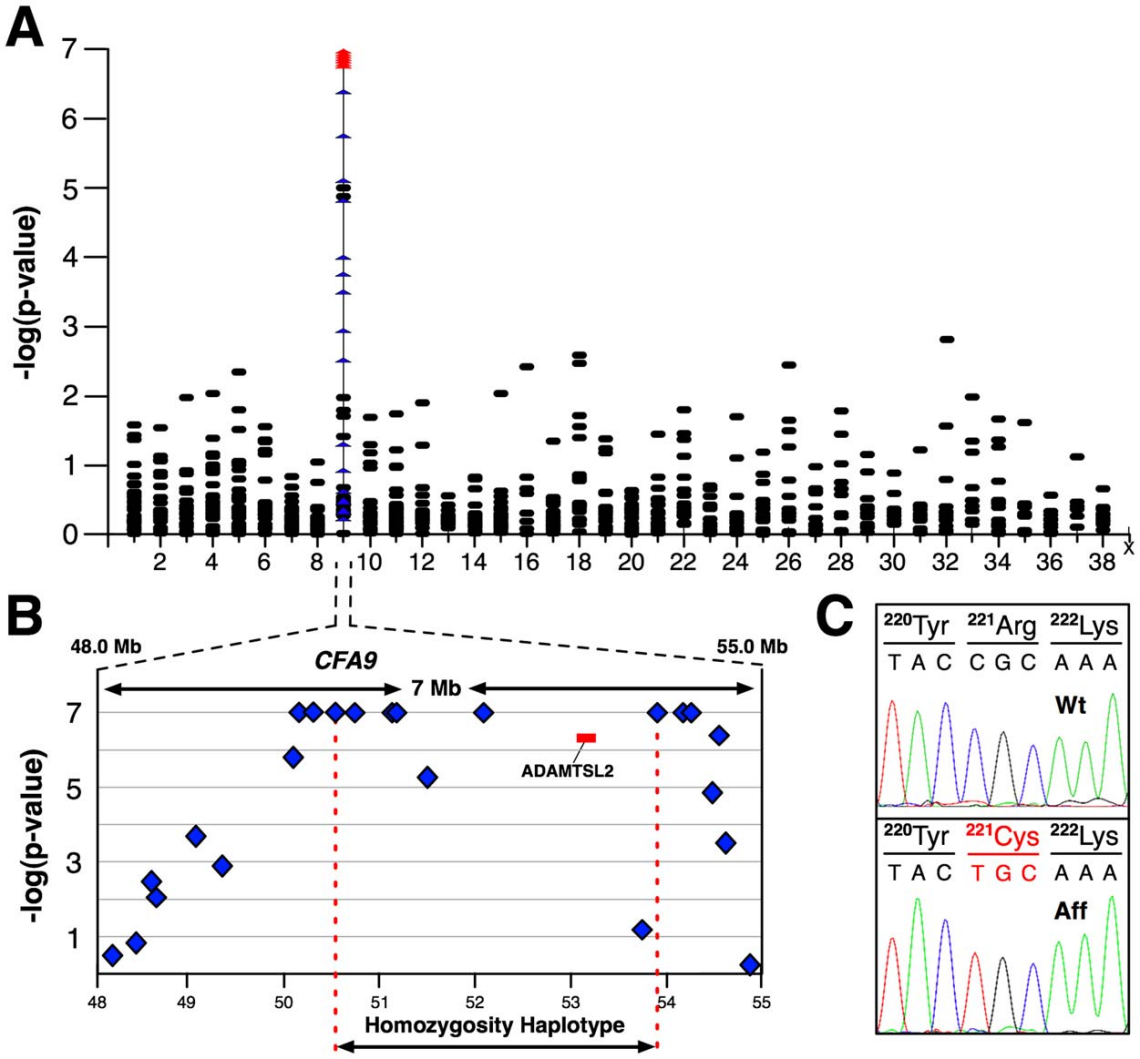
MLS is caused by an *ADAMTSL2* mutation. (**A**) Statistical significance scores from genome-wide case-control analysis of microsatellite-based genotype data are shown. Included are results for loci used in fine-scale mapping of *CFA 9* (n = 23, depicted in blue). Multiple *p*-values estimated by permutation (10^7^ iterations) were saturated for statistical significance at 10^−7^ (red). (**B**) Fine-mapping revealed a six-marker haplotype shared homozygous among all affected dogs (Table S2). The haplotype encompassed a strong candidate gene, *ADAMTSL2* (depicted in red). (**C**) DNA sequence analysis identified the c.660C>T mutation. Sequence traces from PCR products amplified from genomic DNA are shown for a non-affected (Wt) and affected (Aff) Beagle. The transition (C > T) converts an arginine residue to a cysteine (R221C).

To determine the genetic basis of MLS, we applied a set of 719 microsatellite markers (2.6±3.9 cM; 2.8±2.1 Mb) for genome-wide association using a case-control design (6 cases, 39 controls). We found two markers on *CFA 9* that were significantly associated with MLS (*P_raw_* = 10^−5^; **Figure 2A** **and Table S1**). The markers (0934_RD and 0937) were separated by 1.46 Mb (51.10–52.60 Mb). We applied 23 additional CA-repeat loci from the region (*canFam2; CFA 9;* 48.14–54.88*)* for fine-mapping (**Table S2**). Analysis of data generated with an expanded sample set (15 affecteds; 113 unaffecteds) revealed a haplotype (*CFA 9*; 50.11–54.26) that was shared homozygous among all affected dogs (**Figure 2B** and **Table S3**). This suggested inheritance of a founder mutation. Homozygosity was consistent with recessive inheritance. The perfect association of the haplotype was highly significant (p<10^−16^).

### A non-synonymous substitution in *ADAMTSL2* of affected dogs

The critical region contained 112 annotated genes (www.ensembl.org). Two members of the disintegrin and metalloprotease with thrombospondin (*ADAMTS*) gene family (*ADAMTS13* and *ADAMTSL2*) fell within the conserved haplotype. Four members of the ADAMTS superfamily, ADAMTS10, ADAMTS17, ADAMTSL2 and ADAMTSL4 have been implicated in connective tissue disorders, including *ADAMTSL2*, the cause of GD [20], [22], [24], [25]. We sequenced the putative coding regions of both candidate genes (**Table S4**). A single alteration was found in the putative seventh exon of *ADAMTSL2* (**Figure 2C**), which was homozygous for the non-reference allele in an affected dog, and heterozygous in an obligate carrier, consistent with expectations for a causal mutation. This mutation (c.660C>T) predicted a non-synonymous change, converting an arginine to a cysteine at codon 221 (R221C), occurred in a highly conserved stretch of residues, and was computationally predicted to negatively impact protein structure and function (**Figure S1**; *SIFT* [29]). The mutation was confirmed in the DNA of all affected dogs in the study (n = 30). Several carriers were identified, and none showed signs of MLS, consistent with a recessive mode of inheritance.

cDNA from unaffected dog skin obtained by RT-PCR was sequenced to verify the exon boundaries inferred from comparative alignments with the human and mouse orthologs. The *ADAMTSL2* transcript was assembled from overlapping RT-PCR products and Rapid Amplification of cDNA Ends (RACE) analyses (Genbank accession no. GU220770; **Table S5**). The results confirmed the mutation (c.660C>T). *ADAMTSL2* predicted a reading frame of 952 aa, and a translation product of ∼120 kDa. The presence of seven putative N-linked glycosylation sites suggested a slightly greater molecular weight (∼130–140 kDa) than predicted, similar to that previously reported experimentally for mouse ADAMTSL2 [30].

### Effect of the Arg221Cys substition on ADAMTSL2 function

**Figure 3:**
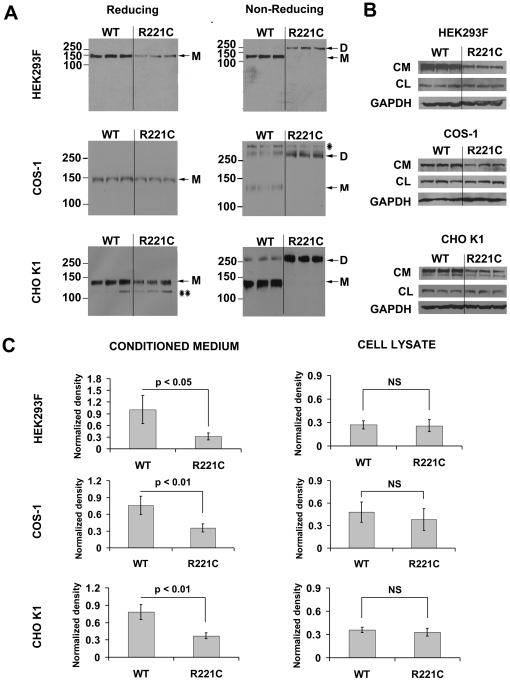
The R221C mutation leads to aberrant ADAMTSL2 dimerization and reduced protein levels. (**A**). Wild-type (WT) or R221C murine ADAMTSL2 was used to transfect HEK293F, COS-1 or CHO-K1 cells (each in triplicate). Conditioned media were run under reducing or non reducing conditions on 6% SDS-polyacrylamide gels, and were analyzed by Western blot using an anti-myc antibody to detect ADAMTSL2. In each cell type, both wildtype and mutant ADAMTSL2 migrated under reducing conditions at 140 kDa, corresponding to the monomeric (M) size of glycosylated ADAMTSL2. The 120 kDa species indicated by a double asterisk in CHO K1 medium, corresponds to an unglycosylated form (confirmed by peptide N-glycanase F digestion, data not shown). Under non-reducing conditions, wildtype ADAMTSL2 migrated predominantly at 140 kDa, although two quantitatively minor species >250 kDa were also observed. The 280 kDa band may correspond to a dimer (D). In contrast, R221C ADAMTSL2 migrated in all 3 cell types exclusively as a 280 kDa dimer (D). (**B**). HEK293F, COS-1 and CHO K1 cells were transiently transfected as described above, and conditioned media and cell lysates were run on 7.5% SDS-polyacrylamide gels under reducing conditions. ADAMTSL2 protein was detected with an anti-myc antibody in both conditioned medium (CM) and cell lysate (CL). In addition, cell lysates were probed with an anti-GAPDH antibody to control for variations in cell numbers. The western blots shown are representative of at least three independent experiments for each cell type. Note that in conditioned media, the level of R221C ADAMTSL2 was reduced compared to wild type ADAMTSL2, whereas the levels in cell lysates were not affected. (**C)**. ECL signal following anti-myc immunoblotting of conditioned media and cell lysates were quantified by densitometry. ADAMTSL2 signal in both conditioned medium and cell lysate was normalized to the GAPDH signal. In conditioned media, the level of mutant protein was significantly reduced, whereas no significant difference was observed in the cell lysates (Student t-test).

We tested for a functional effect by introducing the mutation (c.660C>T) into murine *Adamtsl2* cDNA (R221C ADAMTSL2) and expressing the construct by transient transfection in three cell lines: HEK293F, COS-1, and CHO-K1 (**Figure 3A**–**C**). Conditioned media and cell lysates were analyzed by western analysis with an antibody directed against a C-terminal myc-tag. Under reducing conditions, the expected ∼140 kDa immunoreactive species for both wild-type and R221C ADAMTSL2 was observed in media from each cell line. The same experiments were done under non-reducing conditions to explore the possibility of covalent interactions through the new Cys residue (**Figure 3A**). These experiments revealed an R221C ADAMTSL2 protein species of about twice the molecular weight of wild-type ADAMTSL2 (i.e., 280 kDa instead of 140 kDa) in each of the three cell lines. The contrasting results with reducing and non-reducing analyses implied that the 280 kDa species represented dimers of R221C ADAMTSL2, arising from reducible disulfide bridges formed by the mutant cysteine residues. Low levels of a ∼280 kDa species were also detected in conditioned medium of HEK293F, COS-1 and CHO-K1 cells expressing wild-type ADAMTSL2, suggesting that there may normally be some non-covalent self-association of wild-type ADAMTSL2. In conditioned medium, the signal intensity of the mutant protein was decreased relative to the control transfection in each cell type (**Figure 3B**–**C**). No significant difference was observed in the cell lysates (**Figure 3C**).

## Discussion

Rare human hereditary disorders such as GD often present logistical challenges for study, particularly in recruiting sufficient familial or population cohorts. In contrast, hereditary diseases in the dog sidestep these obstacles, either because crosses can be directed to produce an experimental cohort, or more commonly because inbreeding and favorable pedigree structure (*i.e*., large sibships and multiple generations of extant animals) afford sufficient numbers of affected animals. Another advantage of canine clinical genetics is that allelic and locus heterogeneity is seldom confounding in breed-based mapping. Causal mutations often trace back to a common ancestor within the breed, in many cases to a single animal of foundation stock. Our data suggest this was likely the case for MLS in Beagles. Unique breed predilection suggested the mutation arose within the Beagle gene pool. The disease has been described in England, Australia, America, and Japan, implying an allele age that would afford distribution among broad geographic subpopulations. Accordingly, the mutation probably originated around the time the modern Beagle was being developed, possibly at the end of the nineteenth century. The mutation detection test resulting from this work will assist Beagle breeders in eliminating the mutation from the gene pool. Although allele frequency in the breed is not yet known, the Beagle is one of the more popular breeds with a relatively large population size for purebred dogs. Accordingly, breed diversity need not be negatively impacted by marker-assisted selection. Some breeders may develop a customized strategy to accommodate a high incidence of the mutation in their bloodlines to eradicate the mutation more gradually (*e.g.* continuing to include carriers in the breeding stock, but avoiding crosses that would produce affected offspring).

### Comparative aspects of canine MLS and human GD

Our results show that canine MLS is genetically a counterpart of human GD, a rare disorder whose genetic basis was only recently resolved [20]. Associated phenotypes common to both species include short stature, thick skin, joint contractures, and characteristic facies [19]. A pleasant temperament is reported in humans with GD as well as in dogs, but it has not been formally substantiated in the latter [31]. There are, however, several important features that distinguish the two disorders. In humans, GD can lead to early death stemming from cardiovascular and respiratory complications, which can progress relentlessly over the course of the disease [19]. In contrast, MLS is reported to stabilize after about a year of age and is not lethal. The differences may be attributable to allelic differences, or the canine mutation may have distinct tissue-specific consequences that sidestep cardiovascular and pulmonary effects. Alternatively, genetic and/or physiological background differences between the two species may account for phenotypic variation. Determining the nature of these differences could suggest therapeutic interventions that ameliorate GD, and modify the human disorder toward non-fatal MLS.

Variable expression is reported in both GD and MLS, with much greater variability encountered in GD. The mutations described to date for GD include both nonsense mutations as well as subtle missense changes that appear to result in decreased secretion of ADAMTSL2 [20]. Thus, variable expression of GD could stem from the very different nature of alleles (ranging from nullisomic to hypomorphic mutations), occurring furthermore on a more variable genetic background than in beagles. This is unlikely to explain variable expressivity in canine MLS—an identical mutation was found in all affected dogs and the mutation was characterized on a relatively homogenous breed background.

### Molecular consequences of the ADAMTSL2 mutation in MLS

Previous work showed that mouse ADAMTSL2 mRNA is expressed by skin and perichondrial fibroblasts, and by vascular smooth muscle cells in the lung and large vessels [30]. Tissues containing these cells appeared to be affected which suggests that the role of ADAMTSL2 is local rather than systemic. Indeed, following expression in COS-1 cells, it is localized to the pericellular ECM [30]. The R221C substitution affected ADAMTSL2 by leading to secretion of disulfide-bonded homodimers of mutant ADAMTSL2. In addition, an overall reduction of protein level (i.e. following reduction) was also noted. We speculate that these mutant dimers are non-functional, either in their binding to LTBP1, or to another ECM or cell-surface constituent, with consequent loss of a local regulatory role in TGF-β sequestration or activation. These possibilities will be the subject of future investigations.

### Potential relevance of MLS as an animal model for GD

As an animal model, MLS offers a chance to explore treatments for human GD. Therapeutic intervention is of limited clinical value in the dog because eugenics (*i.e.,* DNA-based selective breeding) has the potential to eliminate the mutation, and thus the disease. MLS dogs may serve as an *in vivo* model for evaluating treatment, since no disease-modifying treatment is currently available for patients with GD. MLS may also provide a model to evaluate therapies aimed at reducing TGFβ signaling in acquired fibrotic diseases, such as pulmonary, hepatic or renal fibrosis, scarring following burns or trauma, and scleroderma. For instance, the angiotensin II receptor (ATIIR)-antagonist losartan was shown to be effective in preventing the progressive aortic dilatation seen in *Fbn1*-deficient mice that was attributed to enhanced TGFβ signaling [32]. In addition, cells and tissues from beagles with MLS may help to elucidate the mechanisms and pathways that normally prevent or attenuate skin and joint fibrosis.

## Methods

### Ethics Statement

The work was conducted with approval of the Animal Care and Use Committee of University of California-Davis (Protocol number #12682).

### DNA and markers

Samples from affected and non**-**affected dogs were recruited through an informal network of private breeders and owners, and through project announcements in breed newsletters and magazines. Ascertainment was of unknown bias, precluding formal analyses of inheritance and heritability. DNA was prepared from blood [33] or buccal swab samples [34] using standard methods. Affected status was established by gross presentation (*i.e*., abnormal gait, joint rigidity, and taut skin). A previously described marker set was used to scan the genome for association [Wong et al, 2008]. The set comprised 719 loci, with an average spacing of ∼5 cM. For fine-resolution mapping, microsatellites were obtained from *DOGSET* [35]. M13-sequence tags were appended to the 5′ end of each forward primer to allow fluorescent label to be incorporated during PCR [36]. A leader sequence (GTTTCTT) was appended to the 5′ end of each reverse primer to catalyze the non-templated enzymatic addition of a nucleotide to the 3′ end of the labeled product strand [37]. Markers were assembled into sets of six loci suitable for multiplex PCR**.** Fine-mapping markers are listed in **Table S2.**

### Genotyping

Microsatellite markers were genotyped using previously described methods (Wong et al, submitted). Fluorescently-labeled PCR products generated from marker-based primer pairs were analyzed using ABI 3730 capillary instruments. The command line option in *GeneMapper4.0* was used to pre-process files remotely. Genotypes were scored with standardized (non-relative) allele assignments consistent across families and populations. All genotypes were manually curated.

### Statistical analyses and computational biology

The software package *Mendel* [38] was used for association mapping. Genotypes of individual markers were tested for differential allele frequency among case and control sample sets. Statistical significance was established by permutation of ‘case’ and ‘control’ labels (10 million iterations). *SIFT* was used to assess the likelihood of a functional effect of the mutation [29].

### Mutation detection

Exons were re-sequenced for polymorphisms with primers obtained from *DOGSET* [35]. T3 and T7 ‘sequence tags’ were appended to the 5′ ends of each forward and reverse primer, respectively, to facilitate high-throughput sequencing. Both strands of each PCR product were sequenced using BigDye Terminator v3.1 (Applied Biosystems) and cycle sequencing. *Phred* [39]was used to assess sequence data quality and *ClustalW* [40] was used to align multiple sequences and identify polymorphisms. Sequence electropherograms were also manually searched for indel polymorphisms.

### cDNA, RT-PCR, and RACE

For transcript analysis, total RNA was isolated from skin biopsy of a male rat terrier with an RNeasy Mini Kit (Qiagen). First-strand cDNA synthesis was performed with Superscript II Reverse Transcriptase (Invitrogen). RT-PCR was performed with primers spanning putative exon boundaries (Supplemental Table S5). RACE was performed with a Marathon Kit (Clontech). PCR products were cloned with a TOPO TA Cloning Kit (Invitrogen). Inserts were amplified by colony PCR of transformants using vector-specific primers (p-topo2.1). Products were sequenced with T3 and T7 primers.

### Cloning, expression, and Western blot analysis

Cloning of murine ADAMTSL2 into the mammalian expression vector pcDNA3.1(−)/myc-his A (Invitrogen) was described previously [30]. The R221C mutation was introduced in this plasmid by site-directed mutagenesis (QuikChange Kit, Stratagene, Thousand Oaks, CA). For transient expression, HEK293F, COS-1 and CHO K1 cells (American Type Culture Collection, Manassas, VA) were seeded in 6 well plates and grown to 80% confluency in DMEM (HEK293F, COS) or MEM-α (CHO-K1) with 10% FBS and antibiotics (100 U/ml penicillin, 100 µg/ml streptomycin). Cells were transfected overnight with 1 µg plasmid/6 µl Fugene 6 (Roche Applied Science, Indianapolis, IN). The next day, the medium was changed from 10% FBS to 1% FBS-containing fresh medium (1 ml/well). After 48 h, conditioned media and cells were collected. Cells were lysed in 100 µl Tris-buffered saline (TBS), 1% Triton X-100 1x EDTA-free complete protease inhibitor cocktail (Roche). Conditioned media (30–50 µl) or the cell lysate were run on a 6% or 7.5% SDS-PAGE under reducing or non-reducing conditions (+/−β-mercaptoethanol). Western blot was performed using a mouse monoclonal anti-myc antibody (9E10, Hybridoma Core, Lerner Research Institute, Cleveland Clinic) at 1∶3000 dilution. A secondary antibody (Jackson Immunoresearch, West Grove, PA) conjugated with horseradish peroxidase was used at 1∶10000. To control for protein loading, western blot of the cell lysate was done using a mouse anti-GAPDH antibody (MAB374, Millipore, Billerica, MA). Blots were analyzed by chemiluminescence using either the ECL Kit (GE Life sciences, Piscataway, NJ) or the Hyglo Kit (Denville Scientific Inc., Metuchen, NJ). For densitometry, the integrated density of bands (pixel x area) was determined with Image J as described in http://lukemiller.org/journal/2007/08/quantifying-western-blots-without.html.

## Supporting Information

Text S1 Clinical and diagnostic information from MLS case studies.
(0.04 MB DOC)

Table S1 Results of genome-wide association mapping with microsatellite markers.
(0.13 MB XLS)

Table S2 De novo microsatellites used for fine-mapping analysis.
(0.02 MB XLS)

Table S3 Results of fine-mapping by case-control association with de novo markers.
(0.01 MB XLS)

Table S4 Primer sequences for amplification and re-sequencing of MLS candidate genes.
(0.04 MB PDF)

Table S5 Primer sequences for RT-PCR and sequence analysis of the ADAMTSL2 transcript.
(0.04 MB PDF)

Figure S1 Amino acid alignment of mammalian ADAMTSL2 proteins.
(0.15 MB PDF)
