# Supplementary material for: An ADAMTSL2 Founder Mutation Causes Musladin-Lueke Syndrome, a Heritable Disorder of Beagle Dogs, Featuring Stiff Skin and Joint Contractures
Source: PLoS One. 2010 Sep 17;5(9):e12817. doi: 10.1371/journal.pone.0012817 (PMC2941456; doi:10.1371/journal.pone.0012817)
Supplement: Text S1 — Clinical and diagnostic information from MLS case studies. (0.04 MB DOC) [file pone.0012817.s001.doc]

**Supplemental File:** Clinical and diagnostic information from MLS case studies.

***Case 1***:

A 4-week old Beagle puppy with MLS was voluntarily submitted by its owner for analysis at the University of Missouri-Columbia Veterinary School. On examination, the affected puppy had the typical facies, thick, immobile skin, short digits, inflexible joints, and tiptoe gait characteristic of the anecdotal reports of the disease. The puppy felt unusually solid, dense, and muscular on palpation. Even under anesthesia, the complete range of motion of stifle and hock joints could not be achieved. The limbs were also notably stiff and rigid.

Echocardiography, electromyography, and nerve conduction analyses were normal. Upon necropsy, the skin showed extensive adhesions to underlying subcutaneous tissues, and superficial tendons, such as the Achilles tendon, were unusually adherent to the skin. No gross anomalies were noted among internal organs: the lungs, heart and cardiac valves, aorta, and brain all appeared normal.

The skull was unusually thick and heavy, although not osteopetrotic. All teeth had erupted on schedule developmentally. Tissue specimens were stained using Hematoxylin & Eosin and the Masson trichrome stain for visualization of collagen. Most tissues *(i.e.*, heart, muscle, lung, heart valve, aorta, spleen, liver) were histologically normal and lacked expected signs of fibrosis. No histological evidence of inflammation was evident. In the skin, extensive fibrosis was seen only in the hypodermis, consistent with the extensive adhesions noted during necropsy.

***Case 2:***

A 4 year old female Beagle that had previously been spayed presented to the Purdue University Veterinary Teaching Hospital (PUVTH) Neurology service for evaluation of an abnormal gait, which had been present since birth. The dog also suffered from bilateral carpus valgus. Other pertinent history included a recent onset of urinary incontinence, and grand mal seizures that started when the case was roughly 2 years of age. Because the seizures had increased in frequency, phenobarbital was prescribed at a dose of 1.76 mg/kg orally every 12 hours. Her gait was characterized by walking on the distal aspect of the digits (“tip-toes”) of all four limbs with rigid extension of all the joints. There was also a carpal valgus deformity in both forelimbs. She had a generalized muscle hypertrophy, including the temporalis muscles, and her eyes were wide set and appeared slanted. Her tail carriage was low and rigid. Upon auscultation, she had a Grade II/VI intermittent left basilar systolic murmur. There were no neurological deficits noted upon examination. Myotatic reflexes were normal, although her flexor withdrawal reflexes were difficult to interpret due to the rigid extension of her limbs.

Initial laboratory analysis included a complete blood count and serum biochemistry profile with creatine kinase (CK). The CBC revealed a panleukopenia and chemistry was unremarkable aside from a mildly elevated ALT (98 IU/L, reference range 3-69) and a mild hypocalcemia (9.5mg/dl, reference range 9.7-12.3). The CK was also within the reference range. The panleukopenia was thought to be secondary to phenobarbital treatment, so the patient was started on levetiracetam (Keppra) at 20mg/kg orally every 8 hours and weaned off the phenobarbital over the course of a week. An ECG revealed a sinus arrhythmia, but no evidence of conduction abnormalities. Echocardiography with Color Doppler revealed turbulent flow across the left and right ventricular outflow tracts; with no valvular changes noted. Spectral Doppler revealed mildly increased pulmonic forward flows during periods of tachycardia. All other cardiac findings were within normal limits. Thoracic radiographs did not show any significant cardiopulmonary changes with a vertebral heart score (VHS, a radiographic tool to evaluate for cardiomegaly) of 12.25, which was considered normal.

A brain MRI was within normal parameters and a cisternal CSF tap did not show any biochemical or cytological imbalances or evidence of inflammation, infectious agents or neoplasia. Electrophysiologic recordings with concentric (coaxial) needles used under general anesthesia revealed mild abnormal spontaneous activity consisting of intermittent positive sharp waves and fibrillation potentials in the semimembranosus/tendinosus, flexors of the carpus, palmar interosseous muscle groups and the muscles of the tail. There was also intermittent increased insertional activity noted in the semimembranosus/tendinosus muscle groups. Motor nerve conduction velocity was performed on the left sciatic-tibial nerve. The conduction velocity was measured at 82.8m/s and the average wave amplitude was 2.9 mV. These findings were within the reference range [1].

Urine was submitted to the Genetics Laboratory at the University of Pennsylvania (PennGen). Metabolic screening revealed a negative mucopolysaccharide spot test and was negative for carbohydrates. The organic acid panel found small hippurate/adipic acid and small amounts of lactate. Amino acid panel revealed an increased glutamate and taurnine. Overall, the screening did not reveal any specific inborn error of metabolism or primary genetic metabolic disorder. The negative MPS spot test excluded a mucopolysaccharidosis disorder.

Serum was submitted to the Infectious Disease Laboratory at the University of Georgia for *Neospora caninum* and *Toxoplasma gondii* titers. The *Neospora caninum* antibody titer (IgG) was 1:64, which is considered a negative result for antibody to Neopsora caninum. Toxoplasa however showed a reciprocal titer (IgM) of 1:1024, which is considered seropositive. The IgG for *Toxoplasma gondii* was 0. This titer elevation was suggestive, but not definitive for, recent exposure to *Toxoplasma gondii*, and convalescent titers were necessary to confirm clinical toxoplasmosis. Because of these results, the dog was treated with clindamycin at a 10mg/kg orally every 12 hours. Follow up *Toxoplasma gondii* antibody titers were negative, indicating that the prior titer was probably not a result of clinical infection. A CBC was repeated at this visit and revealed resolution of her pancytopenia. These findings supported the notion that the pancytopenia was an adverse reaction to the phenobarbital.

1. Walker TL, Redding RW, Braund KG (1979) Motor nerve conduction velocity and latency in the dog. Am J Vet Res 40: 1433-1439.
