# Supplementary material for: An ADAMTSL2 Founder Mutation Causes Musladin-Lueke Syndrome, a Heritable Disorder of Beagle Dogs, Featuring Stiff Skin and Joint Contractures
Source: PLoS One. 2010 Sep 17;5(9):e12817. doi: 10.1371/journal.pone.0012817 (PMC2941456; doi:10.1371/journal.pone.0012817)
Supplement: Table S4 — Primer sequences for amplification and re-sequencing of MLS candidate genes. (0.04 MB PDF) [file pone.0012817.s005.pdf]

**Table S4. Primer sequences for amplification and re-sequencing of MLS candidate genes.**						
**Feature**	**Start (bp)**	**End (bp)**	**Length (bp)**	**Product (bp)**	**Forward Primer**	**Reverse Primer**
ADAMTSL2						
exon 1	53265300	53265428	128	572	cagcaaacacaccaccacct	ctgggtcctgggtcctacag
exon 2	53265981	53266123	142	564	tgggagcaaggacgttaggt	aaacccaggtcaccatcacc
exon 3	53267044	53267119	75	472	gaccccagagaccatttgga	tgagcatctggcacaggact
exon 4	53268207	53268309	102	504	tgttccagacacacgcacag	gctctctgattccccacagg
exon 5	53268814	53268959	145	552	cagagggatgggtcttgtcc	cggtcagtgcaatgagacca
exon 6	53269097	53269220	123	493	tcccactggtctcattgcac	ttaggccccagaatcctcaa
exon 7	53271724	53271804	80	535	gactggccgctcactacctt	agtggccacctacgtgctct
exon 8	53274072	53274247	175	349	acaggtgactggctggcttt	acatgtgggcttcggagact
exon 9	53279110	53279458	348	578	gaggtgcagcatgagtggag	catcccgttcctcctggtta
exon 10	53280980	53281352	372	581	cacacaggggagcatctgag	acggatgctcagccaaactt
exon 11	53286401	53286498	97	447	tgggggagtagagcaaagga	ctgtcagcagcccgtcatag
exon 12	53290631	53290760	129	471	acaaagcaggtgcccagtct	tgagcagaggcaaagcagag
exon 13	53291583	53291796	213	540	tcatcaggtagcagcctcca	gcacagcttctcgtcctcag
exon 14	53291880	53292035	155	600	tccctcactgcttcctcaca	gccacagcaaccacgtttta
exon 15	53292582	53292752	170	477	cggtcctctctgggaatgac	atcatggggcatttggctac
exon 16	53293433	53293609	176	538	ccagttccttcaggcagacc	atgatcactgtggggacgtg
exon 17	53294836	53294980	144	568	cccagggacagtgagagagg	agggaccctggactccattt
exon 18	53295820	53295938	118	509	tccttggcctcctctgatgt	cctggaggcagagaggacag
ADAMTS13						
exon 1	53176830	53176949	119	337	gggaggaagctcccaagagt	gaagaacacccccactctgc
exon 2	53177278	53177344	66	477	tgcttctctcgtctgcttgc	gccagcgcctatcttgtttc
exon 3	53179381	53179418	37	493	atcctgggttgtcgagagga	cgtgctccacttcaggacag
exon 4	53180650	53180810	160	541	ccagccaccactggtcataa	ccactcctcatggtcagcac
exon 5	53182743	53182826	83	497	tttgcatcccgttgtttcag	gtgatgttggccgtgatgtt
exon 6	53182973	53183097	124	582	acctctccctcctgctctcc	tccaggaagatgagcaagca
exon 7	53183220	53183366	146	396	acggcatcactgctgagtgt	ggcctcagcctagaggacaa
exon 8	53184724	53184864	140	542	ccggccaaaggattagtcag	gtggtgggacattcctggtt
exon 9	53185548	53185710	162	508	acctctgtcagccgtgtcct	cgggtgctcacttcactcag
exon 10	53186276	53186380	104	307	tggtgtgtggggaaccatta	aggtgaccaaggtcctctgc
exon 11	53187272	53187423	151	353	gggcattgtcctcttccatc	gacctgaagggcagtgttcc
exon 12	53187543	53187606	63	331	ctgcaacaaccccaggtaca	gcacccccaaatctcaaaag
exon 13	53188960	53189086	126	526	agaccttccctcccgtcttc	atcggggtaatggtggtcag
exon 14	53189485	53189633	148	495	cttccccctactccctgtcc	gaggctctgggaatcacacc
exon 15	53189998	53190118	120	308	tgtggtgtgattcccagagc	catgagggctcagtgtcgag
exon 16	53190643	53190723	80	537	cccaaggaatcccatcagaa	gcaggtggcaccagtagtga

exon 17	53191857	53192038	181	437	tgtgcctatgggaggatgtg	ctgagtcccctaggccctct
exon 18	53192136	53192155	19	335	ctctcccagcaccacctacc	cacccacttccctcaacaca
exon 19	53193742	53193877	135	396	caaggacccgagctctctgt	agcgcaacagcactcactgt
exon 20	53193958	53194087	129	582	aaggtttacaggcgctacgg	atgtgcccacagacaaggtg
exon 21	53194295	53194407	112	347	gctgggctctcactggctac	tgcatcagctctgcactcaa
exon 22	53194635	53194698	63	584	gggaaagtacctggcctgtg	cggtactctgcaccaagcag
exon 23	53194836	53195022	186	463	ggaggccaacactctgttcc	ggacagcaatggcaaagtga
exon 24	53195899	53196078	179	502	cccaccttcctctctggatg	gtgctccccattctcctgac
exon 25	53196727	53196853	126	398	gcaccagcttgttgcttgtc	gctactggggcccttctctt
exon 26	53198000	53198129	129	399	cagagagctggcccctgtag	cagccccacacagtgaacat
exon 27	53198253	53198423	170	492	cctgtccgggaagagctatg	tgtgacccactcttgcaggt
exon 28	53199096	53199278	182	544	agatgaggggaagccctgtt	tgctggcttgagctcttctg
exon 29	53201073	53201277	204	403	caggaatggtgagccattga	actagccccctcctttcagg
exon 30	53201883	53202192	309	487	tcagcctcagcttcctttcc	cagggttctgagggttgctc
exon 31	53202758	53202904	146	589	cctggatggtgacacttgga	ctccaggtgtcctggggtag
exon 32	53203193	53203369	176	399	agtgggtctaccccaggaca	cacacatgctgggacctcat
exon 33	53203675	53203847	172	580	caccaaactttccccсttca	gaggacagtcccccatgaac
exon 34	53204457	53204621	164	509	tatcaaaggctccccgaatg	ctttccagcgagtccagtcc
