# Supplementary material for: An ADAMTSL2 Founder Mutation Causes Musladin-Lueke Syndrome, a Heritable Disorder of Beagle Dogs, Featuring Stiff Skin and Joint Contractures
Source: PLoS One. 2010 Sep 17;5(9):e12817. doi: 10.1371/journal.pone.0012817 (PMC2941456; doi:10.1371/journal.pone.0012817)
Supplement: Table S5 — Primer sequences for RT-PCR and sequence analysis of the ADAMTSL2 transcript. (0.04 MB PDF) [file pone.0012817.s006.pdf]

Table S5. Primer sequences for RT-PCR and sequence analysis of the *ADAMTSL2* transcript.

	ADAMTSL2 cDNA position		Forward primer sequence	Reverse primer sequence	Product
	**Start**	**End**	**(5´-3´)**	**(5´-3´)**	**(bp)**
RT-PCR_1	-1906	240	GGGTGCTCTTTGAAGTGGAG	TGTCCTCCTCTGCTGCAGG	2,146
RT-PCR_2	126	565	CACTGACGCCACTGCCTACT	GATGGGCTCACATTTTCCA	439
RT-PCR_3	222	955	CCTGCAGCAGAGGAGGACA	GTTCTGGTTCCATACCATGACG	733
RT-PCR_4	547	1668	GGAAAATGTGAGCCCATCG	CTGGTCCTGGCCTTGTGAG	1,121
RT-PCR_5	1441	1953	ACGGGTAACAGCATCTTTGC	ACGATGCGGAATTGGTAGC	512
RT-PCR_6	1749	2619	GCACGACAGGGGTCATGT	CAGGTCTTGGTACACTCGGA	870
RT-PCR_7	2266	2619	CGTGCAGTGGAAGCTGTG	CAGGTCTTGGTACACTCGGA	353
