# Supplementary material for: An ADAMTSL2 Founder Mutation Causes Musladin-Lueke Syndrome, a Heritable Disorder of Beagle Dogs, Featuring Stiff Skin and Joint Contractures
Source: PLoS One. 2010 Sep 17;5(9):e12817. doi: 10.1371/journal.pone.0012817 (PMC2941456; doi:10.1371/journal.pone.0012817)
Supplement: Figure S1 — Amino acid alignment of mammalian ADAMTSL2 proteins. (0.15 MB PDF) [file pone.0012817.s007.pdf]

<u>Supplemental Figure S1</u>. *ADAMTSL2* conservation and predicted significance of R221C.

(**A**) Alignment of *ADAMTSL2* orthologs from Ensembl. The mutated arginine (Arg221; red overlay) is among a highly conserved stretch of residues. Sequence similarity among the orthologs listed was 89.7 ± 1.9%. A probable artifact insertion (Ala587) in the Ensembl annotation was revealed by RT-PCR (green overlay). (**B**) Analysis with SIFT [1], a program that assesses the theoretical impact of polymorphisms, predicted R221C was 'probably damaging' (PSIC >2) [2].

A

```
Dog   MDGQRQPSRWVWALLAVVLLAGGVASTGATDNSPTSNSLEGGTDATAYWWGEWTKWTACS 60
Cow   MEGRWQSSRWAWPLLAVALVTGSVSSTGTTDNSPTSNNLEVGPDATAYWWGEWTKWTACS 60
Horse MDGRQWPSHWVWSLLAVLLVAGSAASTGATDNSPTSNSLEGGTDATAYWWGEWTKWTACS 60
Man   MDGRWQCSCWAWFLLVLAVVAGDTVSTGSTDNSPTSNSLEGGTDATAFWWGEWTKWTACS 60
Mouse MDGRRQHPHWAWSLLAVAVVAGGAAPTEASDNSPTSNSLEGGADTTAYWWGEWTKWTACS 60
      *:*:   . *.* **.: :::*.. .* ::*******.** *.*:**:************

Dog   RSCGAGVTSQERHCLQQRRTSVMGAGNRTCTGTSKRYQLCRVQECPPDGRSFREEQCISF 120
Cow   RSCGAGVTSQERHCLQQRKTSVTGAGNRTCTGTSKRYQLCRVQECPPDGRSFREEQCISF 120
Horse RSCGAGVTSQERHCLQQRRTSVTGTGNRTCTGTSKRYQLCRVQECPPDGRSFREEQCVSF 120
Man   RSCGGGVTSQERHCLQQRRKSVPGPGNRTCTGTSKRYQLCRVQECPPDGRSFREEQCVSF 120
Mouse RSCGGGVTSQERHCLQQRRKSVPGTGNRTCVGTSKRYQLCRVQECPPDGRSFREEQCVSF 120
      ****.*************:.** *.*****.**************************:**

Dog   NSRVYDGRTHQWKPLYPDDYVHISSKPCDLHCTTVDGQRQLMVPARDGTSCKLTDLRGVC 180
Cow   NSRVYNGRRHQWKPLYPDDYVHISSKPCDLHCTTVDGQRQLMVPARDGTSCKLADLRGVC 180
Horse NSHVYNGRTHQWKPLYPDDYVHISSKPCDLHCTTVDGQRQLMVPARDGTSCKLTDLRGVC 180
Man   NSHVYNGRTHQWKPLYPDDYVHISSKPCDLHCTTVDGQRQLMVPARDGTSCKLTDLRGVC 180
Mouse NSRVYDGRAYQWKPLYPDDYVHISSKPCDLHCSTVDGQRQLTVPARDGTSCKLTDLRGVC 180
      **:**:** :**********************:******** ***********:******

Dog   VSGKCEPIGCDGVLFSTHTLDKCGVCQGDGSSCTHVTGNYRKGNAHLGYSLVTHIPAGAR 240
Cow   VSGKCEPIGCDGVLFSTHTLDKCGVCQGDGSSCTHVTGNYRKGNTHLGYSLVTHIPAGAR 240
Horse VSGKCEPIGCDGVLFSTHTLDKCGVCQGDGSSCTHVTGNYRKGNTHLGYSLVTHIPAGAR 240
Man   VSGKCEPIGCDGVLFSTHTLDKCGICQGDGSSCTHVTGNYRKGNAHLGYSLVTHIPAGAR 240
Mouse VSGKCEPIGCDGVLFSTHTLDKCGVCQGDGSSCTHVTGNYRKGNNHLGYSLVTHIPAGAR 240
      ************************:******************* ***************

Dog   DIQIVERKKSADVLALADEAGYYFFNGNFKVDSPKNFNIAGTVVKYRRPMDVYETGIEYI 300
Cow   DIQIVERKKSADVLALADEAGYFFFNGNFKVDSPKNFNIAGTVVKYRRPMDVYETGIEYI 300
Horse DIQIVERKKSADVLALADEAGYYFFNGNFKVDSPKNFNIAGTVVKYRRPMDIYETGIEYI 300
Man   DIQIVERKKSADVLALADEAGYYFFNGNYKVDSPKNFNIAGTVVKYRRPMDVYETGIEYI 300
Mouse DIQIVERKKSADVLALADEAGFYFFNGNYKVDSPKNFNIAGTVVKYRRPMDVYETGIEYI 300
      *********************::*****:**********************:********

Dog   VAQGPTNQGLNVMVWNQNGKSPSITFEYTLLQPPHTHRIQPVYYSFSEPTSESAES---Q 357
Cow   VAQGPTNQGLNVMVWNQNGKSPSITFEYTLLQPPHTHRLQPIYYSFSDPTSDSAES---Q 357
Horse VAQGPTNQGLNVMVWNQNGKSPSITFEYTLLQPPHAHRPQPLYYSFSEPAPESTES---Q 357
Man   VAQGPTNQGLNVMVWNQNGKSPSITFEYTLLQPPHESRPQPIYYGFS----ESAES---Q 353
Mouse VAQGPTNQGLNVMVWNQNGKSPSITFEYTLLQSPHMHHLPPVYYSFSEAASQSTESTERQ 360
      ********************************.**  :  *:**.**    :*:**   *
```

Amino Acid Alignment of Mammalian ADAMTS-Like 2 Proteins

```
Dog   ELDGAALLGFLQHNTSFYSQASSERLGLDNRLFGHLGPGIELGLSKGQETNEVCEQAGGR 417
Cow   ELDGAGLAGFLQHNASLFGQASSERLGLDNRLFGHRGPGIELGLSRGQESNEVCAQASGR 417
Horse ELDGADLVGFLQHNGSLYGQASSERLGLDNRLFGHLGPGMELGLPKGQETNEVCEQASGG 417
Man   GLDGAGLMGFVPHNGSLYGQASSERLGLDNRLFGHPGLDMELGPSQGQETNEVCEQAGGG 413
Mouse ELDSARLLGFMQHNGSLYRQTSSERLGLNSQLFQPPAPEVELGPSRGQESNEVCKQASGG 420
       **.* * **: ** *:: *:*******:.:**   .  :*** .:***:**** **.*

Dog   ACEGPPRGKGFRDGNATGTALTGDKDEQQVDKRLTSQRLLSANVISDQLLGAGSDSRELP 477
Cow   ACEGPPRGKGFRDGNATLTALVGDPDDREADARFASQELLSANAISDQLLGAGSDSRELS 477
Horse ACEGPPRGKGFRDGNATGTALAGDQDDHEADVRFASQELLSANAISGQLLGTGSDSKELA 477
Man   ACEGPPRGKGFRDRNVTGTPLTGDKDDEEVDTHFASQEFFSANAISDQLLGAGSDLKDFT 473
Mouse VCEGPPRGKGFQDHNATGRAFSADKDDREISAHFTSHELLSANTISDQLLGTGSESEEFS 480
      .**********:* *.*  .: .* *:.: . :::*:.::***.**.****:**: .::.

Dog   FNETGNSIFAQGAPRRSPAESLYVDYEENEEAGAYLLNGSYLELSSDRVTNSSSEAPFPN 537
Cow   LNETVNSIFSQGAPRGSPAESLYLDYEESEGAGAYLLNGSYLELSSDRLANTSSEAPFPN 537
Horse LNETVNSIFAQGAPRSSPAETFYVDYEENEGAGAYLLNGSYLELSSDRVANTSSEAPFPN 537
Man   LNETVNSIFAQGAPRSSLAESFFVDYEENEGAGPYLLNGSYLELSSDRVANSSSEAPFPN 533
Mouse LNETMNSIFAQGAPRSSPAESLYVDYEENEGPAAYLINGSYLELSSDRIN-TSSEAPFPN 539
      :*** ****:***** * **::::****.* ...**:***********:  :********

Dog   ASSSLPALAGNRTHKARTRPK-TRKQGVSPADMYRWKLSSHEPCSATCTTAGVMSTYAMC 595
Cow   ASASLPALAGNRTQKARTRPK-SRKQGVSPADMYRWKLSSHEPCSSTCTT-GVLSTYAMC 595
Horse ISASLPTTAGNRTHKARTRPKGAASKGVSPADMYRWKLSSHEPCSATCTT-GVMSTYAMC 596
Man   VSTSLLTSAGNRTHKARTRPK-ARKQGVSPADMYRWKLSSHEPCSATCTT-GVMSAYAMC 591
Mouse TSASPPNLAGNRTHKARTRPK-ARKQGVSPADMYRWKLSSHEPCSATCTT-GVMSTYAMC 597
       *:*    *****:******* : .:*******************:****x**:*:****

Dog   VRYDGIEVDDSYCDALTRPEPVQEFCAGRECQPRWETSSWSECSRTCGEGYQFRIVRCWK 655
Cow   VRYDGIEVDDSYCDALTRPEPVQEFCAGRECQPRWETSSWSECSRTCGEGYQFRIVRCWK 655
Horse VRYDGIEVDDSYCDALTRPEPVQEFCAGRECQPRWETSSWSECSRTCGEGYQFRIVRCWK 656
Man   VRYDGVEVDDSYCDALTRPEPVHEFCAGRECQPRWETSSWSECSRTCGEGYQFRVVRCWK 651
Mouse VRYDGVEVDDSYCDALTRPEPVHEFCAGRECQPRWETSSWSECSRTCGEGHQFRIVRCWK 657
      *****:****************:***************************:***:*****

Dog   MLSPGFDSSVYSDLCEAAEAVRPEEHKTCRNPACGPQWEMSEWSECTAKCGERSVVTRDI 715
Cow   MLSPGFDSSVYSDLFEAAEAVRPEERKTCRGPACGPQWEMSQWSECTAKCGERSVVTRDI 715
Horse MLSPGFDSSVYSDLCEAAEAVRPEERKTCRNPACGPQWEMSEWSECTAKCGERSVVTRDI 716
Man   MLSPGFDSSVYSDLCEAAEAVRPEERKTCRNPACGPQWEMSEWSECTAKCGERSVVTRDI 711
Mouse MLSPGFDSSVYSDLCEATEAVRPEERKTCRNPACGPQWEMSEWSECTAKCGERSVVTRDI 717
      ************** **:*******:****.**********:******************

Dog   RCSEDEKLCDPNTRPVGEKDCTGPPCDRQWTVSDWGPCSGSCGQGRMIRHVYCKTSDGRV 775
Cow   RCSEDEKLCDPNTRPVGEKDCTGPPCDRQWTVSDWGPCSGSCGQGRMIRHVYCKTSDGRV 775
Horse RCSEDEKLCDPNTRPVGEKDCTGPPCDRQWTVSDWGPCSGSCGQGRMIRHVYCKTSDGRV 776
Man   RCSEDEKLCDPNTRPVGEKNCTGPPCDRQWTVSDWGPCSGSCGQGRTIRHVYCKTSDGRV 771
Mouse RCSEDEKLCDPSTKPVGEKNCTGPPCDRQWTVSDWGPCSGSCGQGRTIRHVYCKTSDGRV 777
      ***********.*:*****:************************** *************
```

```
Dog   VPESQCQMETKPLAIHPCGDRNCPAHWLAQDWERCNTTCGRGVKKRLVLCMELANGKPQT 835
Cow   VPESQCQMETKPLAIHPCGDKNCPAHWLAQDWERCNTTCGRGVKKRLVLCMELANGKPQT 835
Horse VPESQCQMETKPLAIHPCGDKNCPAHWLAQDWERCNTTCGRGVKKRLVLCMELANGKPQT 836
Man   VPESQCQMETKPLAIHPCGDKNCPAHWLAQDWERCNTTCGRGVKKRLVLCMELANGKPQT 831
Mouse VPESQCQTETKPLAIHPCGDKNCPAHWLAQDWERCNTTCGRGVKKRLVLCMELANGKPQI 837
      ******* ************:**************************************

Dog   RSGPECGLAKKPPEESTCFERPCFKWYTSPWSECTKTCGVGVRMRDVKCYQGTDIVRGCD 895
Cow   RSGPECGLAKKPPEESTCFERPCFKWYTSPWSECTKTCGVGVRMRDVKCYQGTDIVRGCD 895
Horse RNGAECGLAKKPPEESTCFERPCFKWYTSPWSECTKTCGVGVRMRDVKCYQGTDIVRGCD 896
Man   RSGPECGLAKKPPEESTCFERPCFKWYTSPWSECTKTCGVGVRMRDVKCYQGTDIVRGCD 891
Mouse RSGPECGLARKPPEESTCFERPCFKWYTSPWSECTKTCGVGVRMRDVKCYQGTDIVRGCD 897
      *.*.*****:**************************************************

Dog   PLVKPVGRQACDLQPCPTEPPDDSCQDQPGTNCALAIKVNLCGHWYYSKACCRSCRPPHS 955
Cow   PLVKPVGRQACDLQPCPTEPPDDSCQDQPGTNCALAIKVNLCGHWYYSKACCHSCRPPPS 955
Horse PLVKPVGRQACDLQPCPTEPPDDSCQDQPGTNCALAIKVNLCGHWYYSKACCRSCRPPHS 956
Man   PLVKPVGRQACDLQPCPTEPPDDSCQDQPGTNCALAIKVNLCGHWYYSKACCRSCRPPHS 951
Mouse PLVKPVGRQACDLQPCPTEPPDDSCQDQPGTNCALAIKVNLCGHWYYSKACCRSCRPPHS 957
      ****************************************************:***** *
```

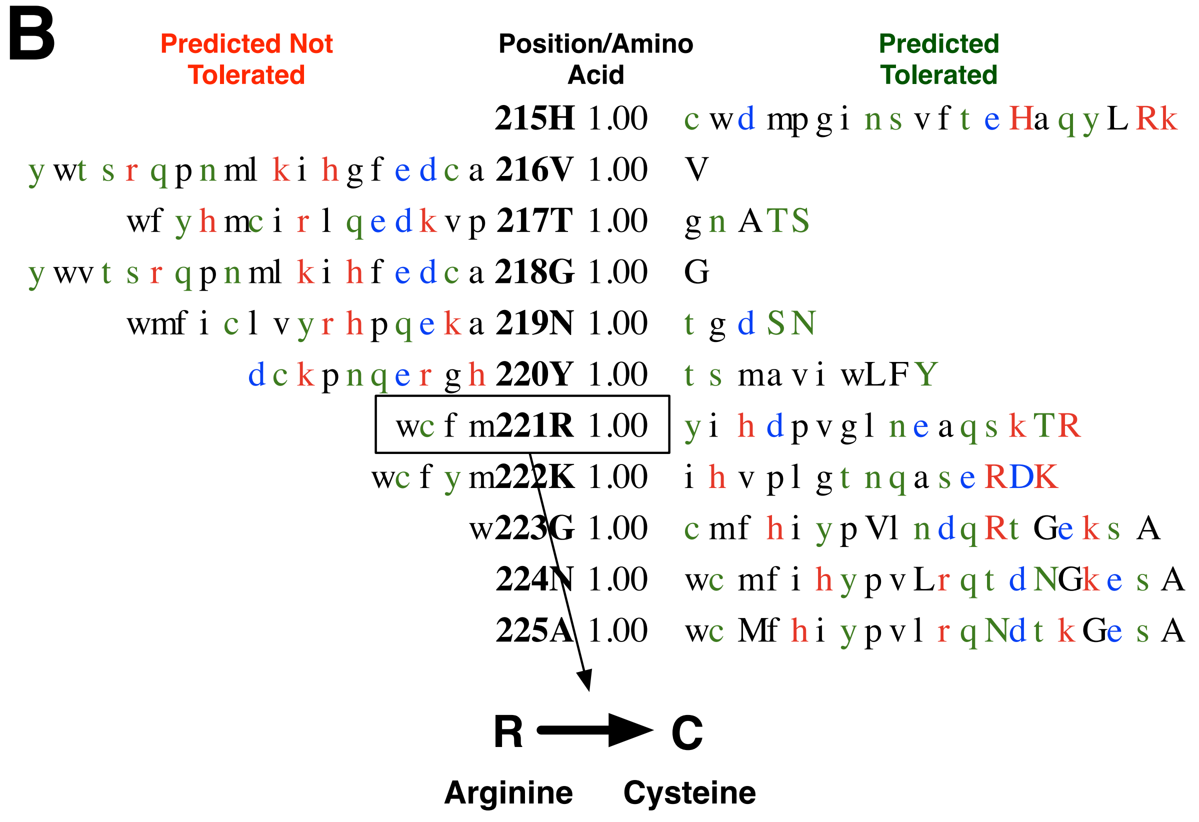

Substitution at pos 221 from R to C is predicted to AFFECT PROTEIN FUNCTION with a score of 0.03. Median sequence conservation: 3.05 Sequences represented at this position:7

[1] Ng PC, Henikoff S (2003) SIFT: predicting amino acid changes that affect protein function. *Nucleic Acids Res*. 31:3812-3814.

[2] Sunyaev S, Ramensky V, Koch I, Lathe W 3rd, Kondrashov AS, Bork P (2001) Prediction of deleterious human alleles. *Hum Mol Genet*. 10:591-597.
